# Piloting a scale-up platform for high-quality human T-cells production

**DOI:** 10.3389/fcell.2024.1427171

**Published:** 2024-07-12

**Authors:** Viknesvaran Selvarajan, Denise Bei Lin Teo, Chaw-Chiea Chang, Yuen Ling Ng, Nge Cheong, Jaichandran Sivalingam, Soo Hean Gary Khoo, Adison Wong, Bernard Liat Wen Loo

**Affiliations:** ^1^ Food, Chemical and Biotechnology, Singapore Institute of Technology, Singapore, Singapore; ^2^ Chemical Engineering, Newcastle University in Singapore, Singapore, Singapore; ^3^ Quintech Life Sciences Pte. Ltd., Singapore, Singapore; ^4^ Tessa Therapeutics Ltd., Singapore, Singapore

**Keywords:** bioprocessing, adoptive cell therapy, stirred-tank bioreactor, biaxial rotary bioreactor, scale-up, T-cell

## Abstract

Cell and gene therapies are an innovative solution to various severe diseases and unfulfilled needs. Adoptive cell therapy (ACT), a form of cellular immunotherapies, has been favored in recent years due to the approval of chimeric antigen receptor CAR-T products. Market research indicates that the industry’s value is predicted to reach USD 24.4 billion by 2030, with a compound annual growth rate (CAGR) of 21.5%. More importantly, ACT is recognized as the hope and future of effective, personalized cancer treatment for healthcare practitioners and patients worldwide. The significant global momentum of this therapeutic approach underscores the urgent need to establish it as a practical and standardized method. It is essential to understand how cell culture conditions affect the expansion and differentiation of T-cells. However, there are ongoing challenges in ensuring the robustness and reproducibility of the manufacturing process. The current study evaluated various adoptive T-cell culture platforms to achieve large-scale production of several billion cells and high-quality cellular output with minimal cell death. It examined factors such as bioreactor parameters, media, supplements and stimulation. This research addresses the fundamental challenges of scalability and reproducibility in manufacturing, which are essential for making adoptive T-cell therapy an accessible and powerful new class of cancer therapeutics.

## Introduction

Adoptive cell therapy (ACT) represents a revolutionary approach to personalized immunotherapy for human cancer, viral infection and autoimmunity (Rosenberg Steven and Restifo Nicholas, 2015; Depil et al., 2020). This technique involves the infusion of a patient’s immune cells (autologous) or another individual (allogeneic) to target and eradicate tumor cells, offering a highly customized treatment option (Feins et al., 2019). One of the most promising forms of ACT is chimeric antigen receptor (CAR) T-cell therapy. CAR-T cell therapy modifies T-cells to express receptors specific to cancer antigens, enabling these engineered cells to recognize and destroy malignant cells with remarkable precision (Feins et al., 2019). However, developing “off-the-shelf” allogeneic CAR-T cells derived from healthy donors rather than the patient presents unique challenges. These challenges include ensuring the cells’ compatibility with the recipient’s immune system and avoiding graft-versus-host disease (GvHD), which remains a significant barrier (Depil et al., 2020). Despite these obstacles, advances in targeting strategies and the identification of safe targets are paving the way for more effective and widely applicable therapies, enhancing the potential of ACT as a powerful potential in cancer treatment (Hammerl et al., 2018).

There has been a paradigm change in the treatment of aggressive B-cell lymphoma and acute lymphoblastic leukemia. The current Food and Drug Administration (FDA) approval of the first chimeric antigen receptor T-cell products (CAR-T), the chimeric Kymriah^®^ (tisagenlecleucel) and Yescarta^®^ (axicabtagene ciloleucel) has resulted in remissions of up to 90% of treated patients with hematologic malignancies (Sadelain, 2017).

CAR-T therapies have shown promise in revolutionizing cancer treatment, yet their usage is presently constrained to addressing patients with selected liquid tumors at the relapsed and refractory stages. CAR-T entails genetically modifying T-cells from the patient or a donor to express a chimeric antigen receptor that targets a specific tumor antigen (Tang et al., 2018). The *ex vivo* production of T cell-based ACTs typically includes (a) isolating mononuclear cells or enriched T-cells from patient blood or tissues; (b) activating the cells; (c) genetically modifying them (using viruses, transposons or CRISPR); (d) expanding the cells and (e) formulating the cell product (June et al., 2018). These processes may require several weeks to months to finish, resulting in high expenses and patient deterioration (Levine et al., 2017; Iyer et al., 2018). The remarkable worldwide momentum of this therapeutic strategy underscores the pressing requirement to establish it as an efficient and standardized method.

Expansion of T-cells is a pivotal stage in numerous cellular immunotherapy protocols, demanding significant quantities of immune cells to eliminate malignant cells. These cellular therapies demand the production of over ≥10^9^ autologous or allogeneic T-cells for patient infusion. Therefore, technologies that can streamline processes, minimize labor and cut costs while adhering to Good Manufacturing Practice (GMP) standards are highly preferred. Nonetheless, it continues to pose a significant translational and commercial challenge due to the manual, small-scale and frequently static culturing systems employed in their production (Aijaz et al., 2018). Cell therapy manufacturing also faces a plethora of challenges that can ultimately restrict the treatable patient population and result in unpredictable and incredibly variable clinical results. Scientists are turning to advanced gene editing techniques to consistently and more affordably modify patients’ cells to express chimeric antigens, cell receptors and other molecules with enhanced effectiveness and deactivate or alter genes more efficiently (Ying et al., 2019). The main concern is that primary T-cells are sensitive to shear forces, which can affect their relevant phenotypes and consequently hinder their growth in more scalable and dynamic systems such as stirred-tank bioreactors and the WAVE bioreactor when compared to static conditions (Costariol et al., 2019). Nonetheless, utilizing stirred culture systems for T-cell expansion presents numerous potential benefits over the current static culture systems, including uniformity in culture conditions, simplified sampling procedures and the ability to implement control systems. WAVE bioreactors are suitable for the scale-up of T-cell expansion for cell therapy applications due to their scalability, automation, closed system design, efficient mixing capabilities, and flexibility for use with various cell types and applications. These features of both systems help to ensure consistency, reproducibility, and sterility in the culture environment, which are critical for the production of T-cells for cell therapy.

The existing manufacturing processes for cell and gene therapies predominantly rely on manual, planar culture systems. These methods are labor-intensive and frequently involve open processes challenging to scale up, relying significantly on the operator’s expertise and discretion. Consequently, they are susceptible to human error, increasing batch-to-batch variability, high production costs, and an elevated risk of contamination and batch failure. The development of advanced therapies, particularly in adoptive cell therapy, has shown significant promise in treating various diseases. The expansion of lymphocytes in bioreactors represents a crucial advancement, enabling the mass production of these therapeutic cells, which is essential for effective treatment delivery (Garcia-Aponte et al., 2021). Addressing the pressing needs in this domain involves overcoming numerous challenges, including scaling up production and guaranteeing the safety and effectiveness of these therapies (Morrow et al., 2017). Recent advances, such as the development of allogeneic CAR-T cells, have further propelled the field, offering new possibilities for off-the-shelf treatments that can be administered to multiple patients without needing patient-specific cell modifications (Kim and Cho, 2020).

The recent achievements in cell therapy utilizing CAR-Ts underscore the revived attention towards bioprocessing and the manufacturing facilities necessary to fulfill the demands by expanding cultures with bioreactors. This study explores the growth of primary T-cells sourced from healthy human donors. It illustrates the application of adaptable bioreactor platforms for manufacturing T-cells that preserve functionality and transition to suitable phenotypes for cancer immunotherapy. Furthermore, we demonstrate the feasibility of achieving this at a smaller scale (0.5 L), consistently producing several billion CD3^+^ T-cells within a short timeframe, thereby offering the potential for substantial reductions in labor and expenses through a methodical process development approach.

### Experimental design

#### T-cell isolation

Peripheral blood mononuclear cells (PBMCs) were supplied by Stemcell Technologies, Vancouver, Canada and obtained from healthy donors who provided written informed consent. The acquirement of PBMCs was carried out using approved consent forms and protocols in accordance with the Institutional Review Board (IRB). CD3^+^ T-cells were isolated using EasySep™ Human T-cell Isolation kit following the manufacturing instructions. Cryopreserved PBMCs were thawed and washed twice with EasySep buffer (Ca^2+^ and Mg^2+^ free PBS, 2% FBS, 1 mM EDTA, pH 7.4) first. Cells were then incubated with DNase I solution at room temperature for 15 min to disrupt aggregated suspension cells. Isolation Cocktail (Stemcell Technologies, Vancouver, Canada) was added and incubated at room temperature for 5 min, followed by magnetic spheres. The cell suspension was placed in a magnetic holder to capture CD3^+^ T-cells, creating a high-gradient magnetic field that effectively separates cells labeled with magnetic particles without requiring columns. The magnet is then lifted with the tube and inverted, and the isolated cells are collected in a centrifuge tube. The untouched T-cells underwent two washes with culture medium before activation.

#### T-cell stimulation and cell culture

The isolated CD3^+^ T-cells were seeded at one million cells/mL in 6-well plates for stimulation. ImmunoCult™ Human CD2/CD3/CD28 T-cell Activator (Stemcell Technologies, Vancouver, Canada) was added at 25 μL/mL culture and cultured in basal media, RPMI-1640 supplemented with 10% FBS (Capricorn Scientific GmbH). The T-cell cultures were supplemented with 50 IU/mL IL-2 from Day 0.

The T flask static cultures were maintained at 37°C, 5% CO_2_. AppliFlex ST (Applikon Biotechnology B.V., Netherlands), a fully customizable 3D printed single use stirred-tank 0.5 L bioreactor, was controlled at 37°C, pH 7.3, dissolved oxygen (DO) 100%, agitation 200 revolutions per minute (rpm) and gas sparging rate 0.01 volume of liquid per minute (VVM). Tisxell Regeneration System (Quintech Life Sciences, Singapore) is unique in its bi-directional revolution. The 0.5 L biaxial bioreactor rotates along two separate axes, enabling it to spin and tumble simultaneously or operate continuously. Both axes were regulated to operate as a unified axis system with agitation set at 5 rpm. Single use 2 L cell bags were used on the ReadyToProcess WAVE™ 25, rocking bioreactor system (Cytiva, United States). Cellbags were aerated with 5% CO_2_ and agitated by rocking at 20 rpm with a 5°angle, pH was set to be maintained at 7.3 and DO was set to be at 100%. Temperature was maintained at 37°C. The pH of all the cultures in reactor culture systems was adjusted with 1 M Sodium Bicarbonate. Inoculation was done at a viable cell density of 1.25 × 10^5^ cells/mL in all culture systems. Daily samples were drawn sterilely from the needleless ports.

#### Analytical techniques

Daily samples were collected from the cell expansion cultures to monitor cell growth, measuring cell density and viability with the NucleoCounter^®^ NC‐200™ (ChemoMetec A/S^©^, Denmark) using the Via 1‐Cassette™ (ChemoMetec A/S^©^, Denmark), which contains acridine orange and DAPI. Subsequently, the same sample was analyzed using the Cedex Bio Analyzer (Roche Diagnostics GmbH, Germany) to determine glucose, glutamine, ammonia and lactate concentrations.

#### Flow cytometry analysis

Antibody panel used in this study were purchased from Miltenyi Biotec GmbH, Germany, and consist of anti-CD25 (PE-Vio770), anti-CD69 (APC), anti-CD4 (PerCP-Vio^®^ 700), anti-CD8 (PE), anti-CD45RO (PE-Vio615), anti-CD45RA (APC), anti CD197 (PE-Vio770), anti-PD1 (PE-Vio770), anti-LAG3 (APC), anti-TIM3 (PE-Vio615), anti-IFN-γ (APC), anti-IL-2 (PE-Vio770), and anti-IL-4 (PE). CD3-UCHT1 (FITC) was obtained from Stemcell Technologies Inc., Canada. The staining dyes were selected based on their fluorescent compatibility and the analyzer’s capabilities. Cell surface staining was done by harvesting cells and washing them with a staining buffer (PBS, 0.5% BSA, 2 mM EDTA) at 400 g for 5 min. Cells were stained with relevant antibodies and Fixable Viability Stain 780 (BD Biosciences, United States), incubated in the dark at 4°C for 10 min, and washed with staining buffer before analysis. The Inside Stain kit (Miltenyi Biotec GmbH, Germany) was used for intracellular staining. Cells were fixed using Inside Fix solution and incubated for 20 min in the dark before co-staining with relevant surface markers. The fixed cells were washed with Inside Perm Buffer, then stained with anti-IFN-γ, anti-IL-2 and anti-IL-4 antibodies in the dark at 4°C for 10 min. After staining, the cells were washed twice and re-suspended in the staining buffer for analysis. The expression of surface and intracellular markers in the T-cell samples was analyzed using the Guava^®^ easyCyte™ flow cytometer (Luminex Corporation, United States). The data were processed with FlowJo software (FlowJo LLC, United States). Live cells and singlets were gated before performing the analysis. Gating was established, so the negative sample exhibited less than 0.5% of the fluorescent population.

#### Statistical analysis

Experimental data was analyzed using GraphPad Prism nine software (GraphPad, La Jolla). The results are presented as mean ± SD. Cell function and expansion capability were analyzed to compare across donors. A one-way analysis of variance (ANOVA) test was utilized and a *p*-value of ≤ 0.05 was deemed statistically significant for all tests.

## Results

### Development and optimization of a scalable T-cell expansion process

**FIGURE 1:**
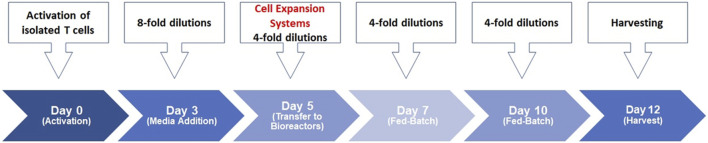
Human T-cell expansion timelime. T-cells were stimulated on day 0 using ImmunoCult™ Human CD3/CD28/CD2 T-cell Activator in ImmunoCult™-XF T-cell Expansion Medium supplemented with IL-2 in static culture. Total culture volumes were increased by 8-fold using fresh medium on day 3. Total culture volumes were further increased by 4-fold on day 5 and transferred to the respective bioreactor systems. Basal media was added to the cultures by 4-folds on day 7 and day 10 until harvest on day 12 of the process.

We previously described our scale-up process for T-cell activation (Selvarajan et al., 2020; Selvarajan et al., 2021). CD3^+^ T-cells were isolated from healthy donors’ PBMCs and were then stimulated with soluble antibody complexes, providing the signals needed for activation. The cells were incubated in static culture vessels for 5 days before being processed in static and bioreactor expansion systems, carefully controlling temperature, pH, dissolved oxygen, agitation and gas sparging. Daily samples from the bioreactor cultures monitored cell growth and quality throughout T-cell production. The early stage involves thymocyte positive selection, the middle stage involves differentiation into CD4^+^ or CD8^+^ T-cells and the late stage involves mature T-cells migrating to peripheral lymphoid organs and activating upon encountering foreign antigens (Cavalheiro et al., 2017). This study established a bioreactor-based human T-cell expansion platform by assessing various cell culture factors, including basal media, medium supplements, seed culture, stimulation strategy, scale-up, and donor variability. The streamlined cell expansion timeline is depicted in Figure 1; human T-cells were purified and activated on Day 0, expanded into bioreactor systems on Day 5, and harvested 12 days later.

### Comparative analysis of T-cell expansion in various bioreactor systems

**FIGURE 2:**
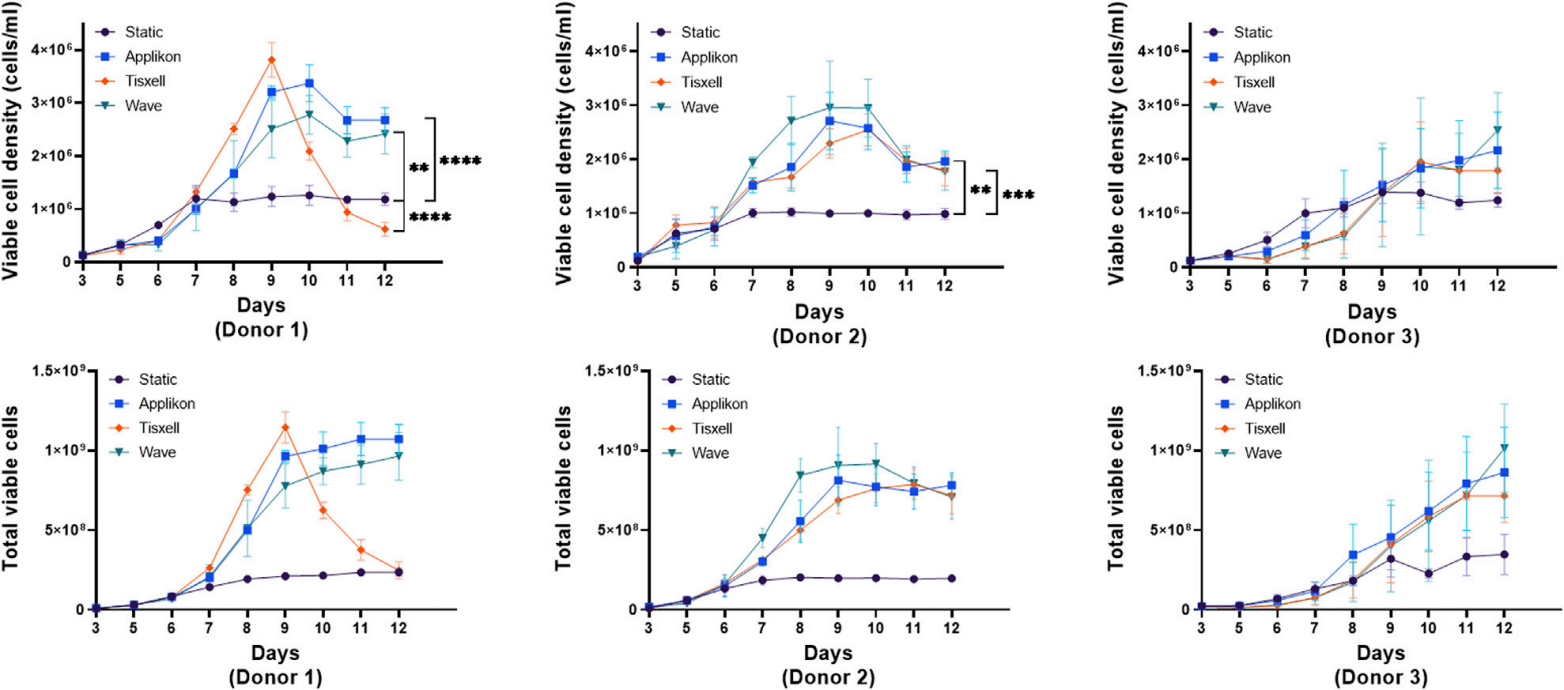
T-cell growth kinetics in bioreactors. The growth of human T-cells from independent donors was compared against the bioreactors over 9 days is displayed. Cells were seeded equally for three healthy donors with high viability (∼98%). All donors had variable expansion profiles across the bioreactors. Appliflex and WAVE bioreactors showed elevated fold expansion (fold change: 31–36) compared to the static control on harvest. Tisxell bioreactor had only a marginal increase due to early growth saturation for Donor 1. All bioreactors for Donor two were uniformly expanded (fold change ∼20) relative to the control T-flasks. There was no significant growth improvements for Donor three in comparison to the static cultures. Data are shown as mean ± SD. Statistical significance is indicated in the plots if applicable, *p* ≤ 0.05 (*), *p* ≤ 0.01 (**), *p* ≤ 0.001 (***) or *p* ≤ 0.0001 (****).

High viable (98%) CD3^+^ enriched T-cells from three healthy donors were seeded in the culture systems at 25 × 10^6^ per vessel. Indicative seeding numbers of T-cells were utilized to examine the versatility of the Applikon, Tisxell, and WAVE bioreactor systems compared to the static T flask condition for expanding T-cell products. Figure 2 illustrates the expansion and growth kinetics of cells from individual donors. Our human T-cell platform’s expansion was systematically assessed through static T flasks and scaled-up bioreactor cultures. The seed cultures were kept consistent on all donors up to day 5 post T-cell activation. The higher viable cell density in the bioreactors is due to the enhanced oxygen transfer rate. Secondly, we expanded T-cells in bioreactors with a working volume of 400 mL. Utilizing automatic process control for pH, DO and gas sparging, we achieved a maximum viable cell density (VCDmax) of 2.4 × 10^6^ cells/mL. Our data showed the donor-dependent variability of cell expansion between donors. The stirred-tank Appliflex and WAVE systems showed a 31–36-fold expansion compared to the static culture on the harvest. The final harvested cell products for the Tisxell biaxial rotary bioreactor were marginal due to early growth saturation. It clearly shows the different growth environments in the different bioreactors, and for donor 1, similar folds of cell expansion could be achieved even on day 9 in the Tisxell bioreactor. It is important to note that the Tisxell bioreactor lacks control for oxygen and pH. These data demonstrate that we can scale up the established bioreactor-based platform to produce a large number of T-cells. All culture systems for donor two were less expanded (20-fold) relative to the control T flasks. T-cells were not significantly expanded for donor 3 after the scale-up. The robustness and reproducibility of this process, demonstrated by cultivating T-cells from three donors, provide a foundation for further investigation.

**FIGURE 3:**
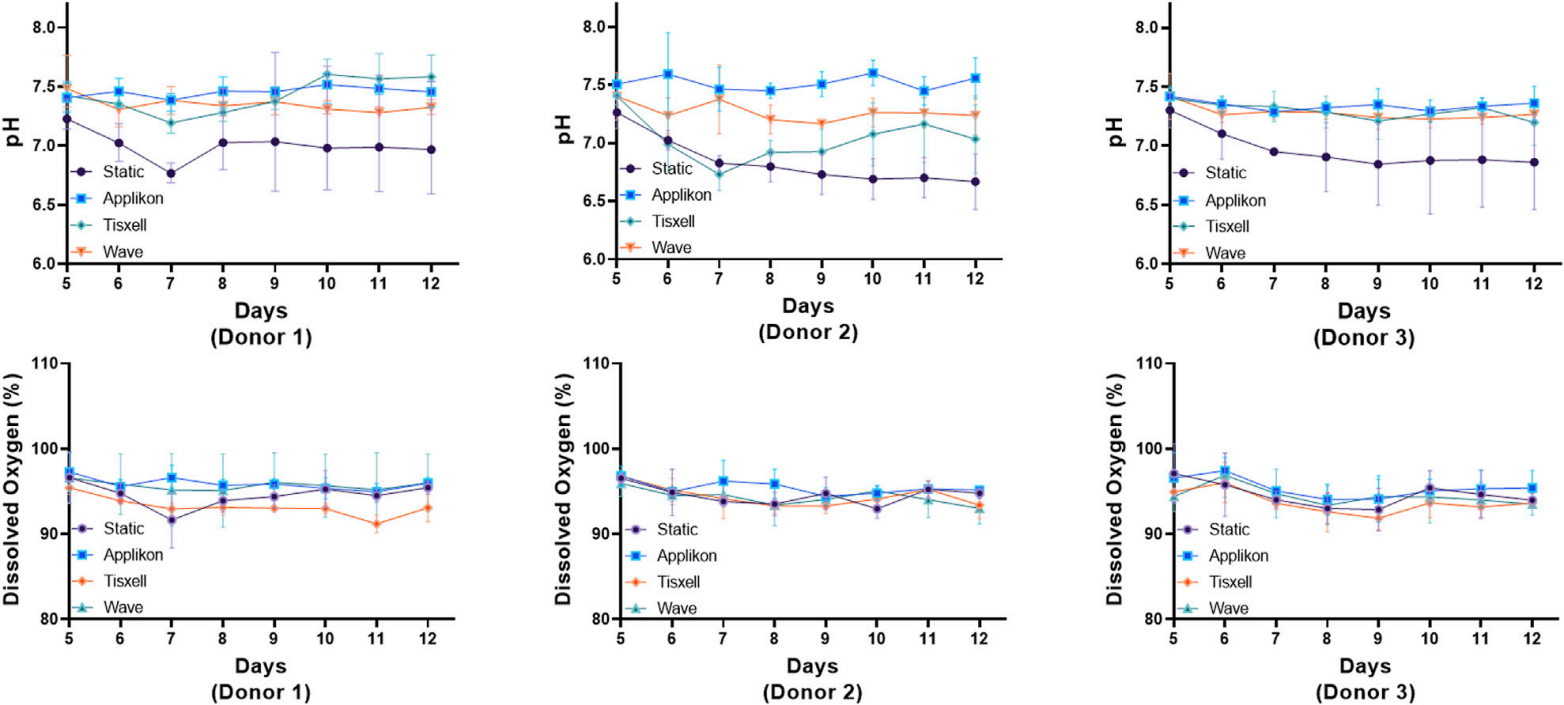
pH and dissolved oxygen (DO) trends under various bioreactors. The Applikon and Wave reactors had automatic process control of pH and DO (gas sparging). The pH profiles aligned with the viable cell concentration and with the exhaustion and yield of metabolites. DO remains stable >95% throughout the culture duration while matching the oxygen demand for cell growth. The subtle spikes observed in both pH and DO are due to media addition on respective days. Data are shown as mean ± SD.

**FIGURE 4:**
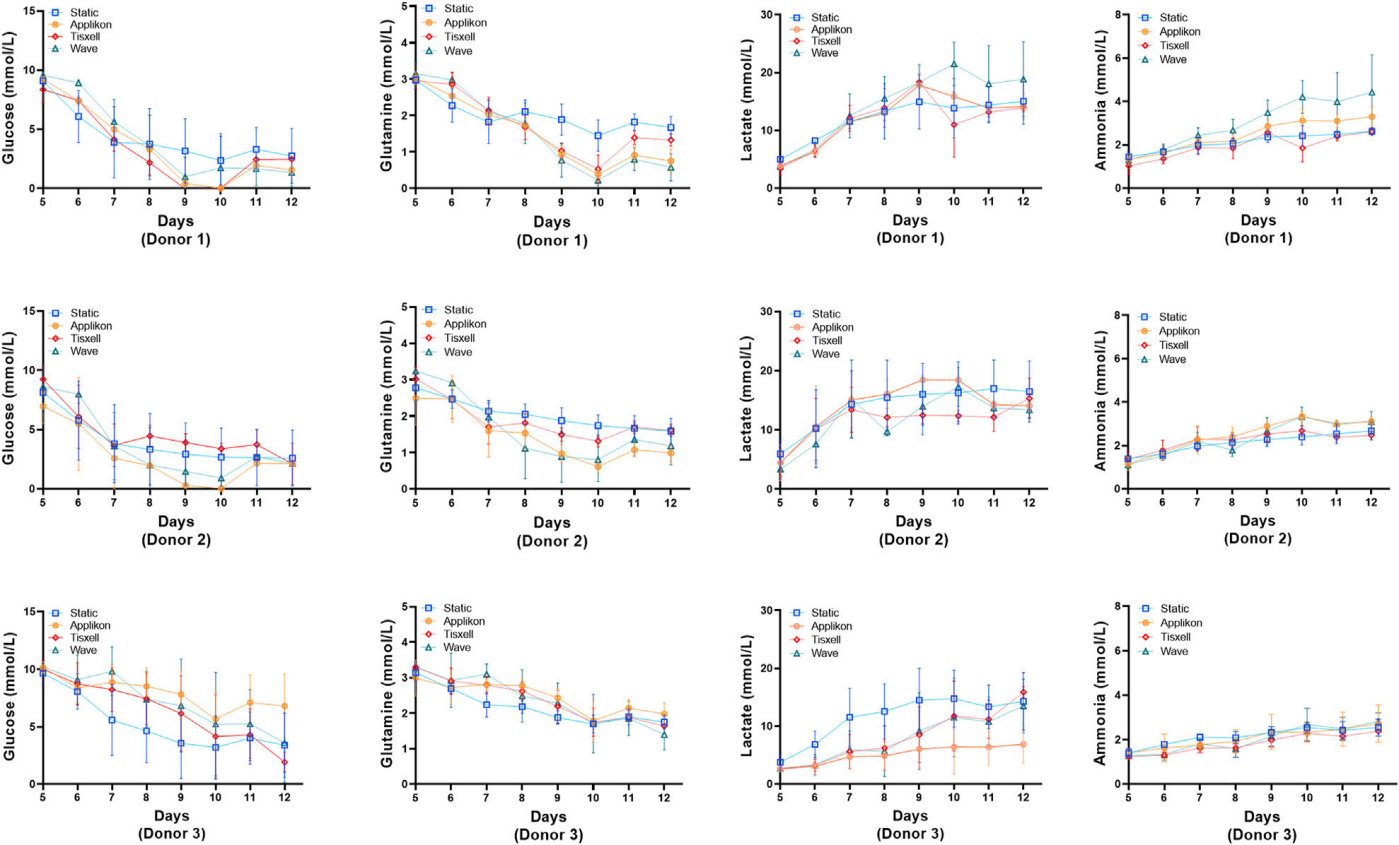
Metabolite concentrations for the bioreactors. Levels of glucose, glutamine, lactate and ammonia were monitored daily. Glucose and glutamine consumption correlates well with the cell growth kinetics data. Lactate and ammonia production reflects the expansion cell growth trend. Lactate generation peaked from Day 7 followed by rapid decline of glucose and glutamine. Data are shown as mean ± SD.

The pH profiles conformed with the viable cell concentrations and were aligned with cell exhaustion and yield of metabolites. DO remains stable (>95%) throughout the culture duration while matching the oxygen demand for cell growth (Figure 3). Glucose and glutamine consumption and lactate and ammonia production rates were monitored offline daily starting on Day 5 and are presented in Figure 4. Glucose and glutamine consumption correlates well with cell growth kinetics. The concentrations indicate a progressive decline observed during the exponential cell growth in all culture systems across all donors. Elevated profiles for lactate production were observed from seed culture transfer on Day 5, indicating aerobic glycolysis. As commonly observed in cell culture, the experimental concentrations were within the typical range, which is not inhibitory (Quinn et al., 2020). Ammonia, a byproduct of glutamine metabolism, exhibited a modest increase within acceptable limits for all donors (Hassell et al., 1991).

### Process characterization and quality assessment of expanded T-cells

**FIGURE 5:**
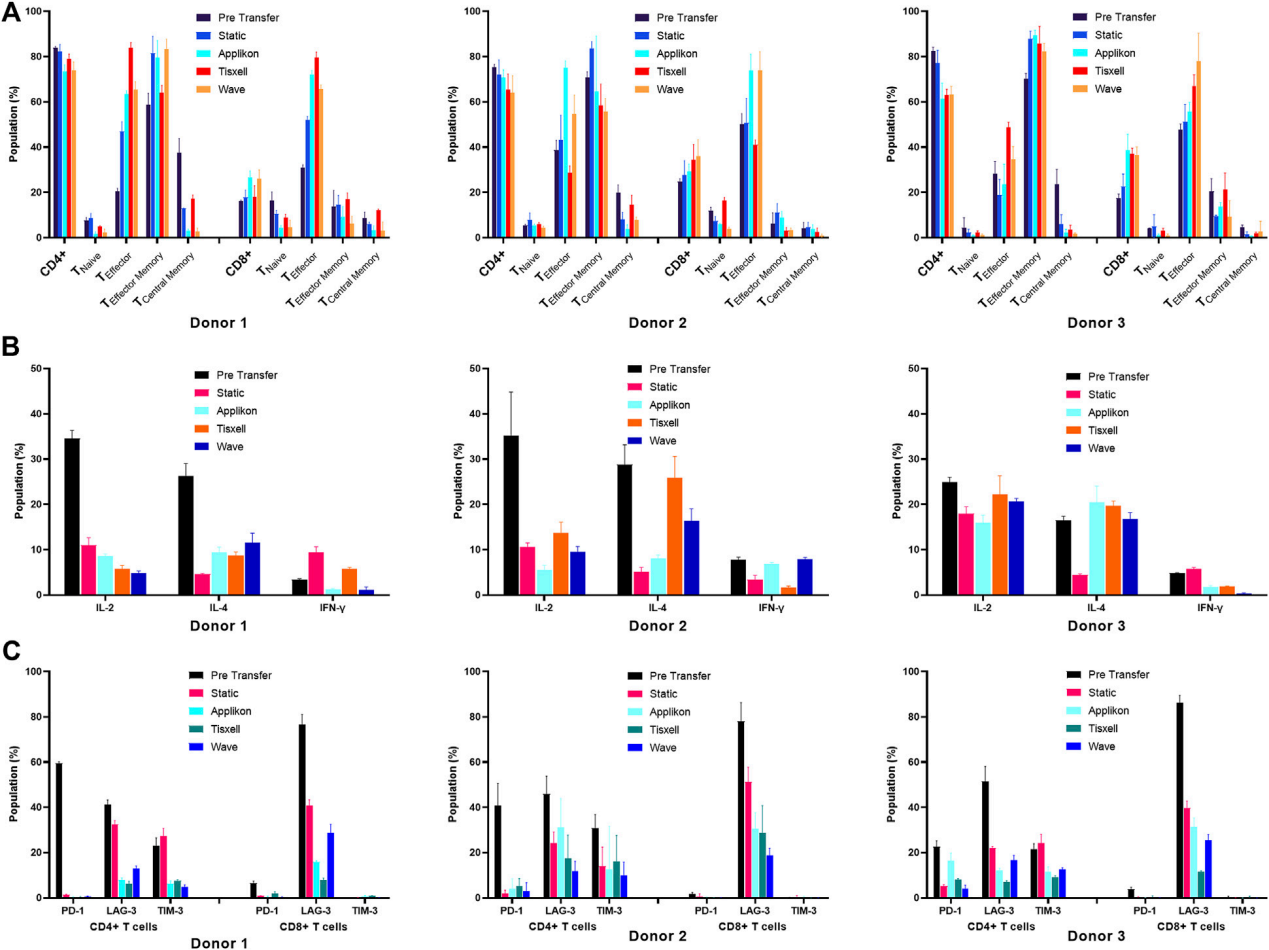
Phenotype characterization of T-cells. **(A)** T-cell differentiation Surface markers, **(B)** Exhaustion markers and **(C)** Intracellular cytokines. T-cells subpopulations are analyzed pre-transfer and post-harvest on all culture systems. Cell populations are gated based on isotype controls. Cell quality was assessed based on CD4^+^ and CD8^+^ T-cell subpopulations. **(A)** CD4 to CD8 T-cell ratio was taken as a quality indicator of the final yields. Ratio (CD4:CD8) was higher (5:1) before transfer and reduced (2:1) as the expansion progressed. Expression profiles of the memory cell types had increased progressively for all donors. **(B)** Potential T-cell exhaustion was assessed in the harvested cell products. The upregulated inhibitory markers (PD-1 and LAG-3) on pre-transfer indicate the poor proliferation abilities of the cultivated T-cells. It was noted that collective suppressive markers decreased significantly along with the cell expansion. **(C)** T-cell function was evaluated by measuring the levels of intracellular cytokines. T-cells secreted high amounts of pro-inflammatory (IL-2 and IFN-γ) and anti-inflammatory (IL-4) cytokines upon stimulation (pre-transfer). Harvested T-cells had greatly reduced background levels across all donors. Data are shown as mean ± SD.

Process characterization is essential for providing scientific evidence that the procedure consistently produces high-quality adoptive cell therapy products. It typically involves verifying product identity, purity, safety, and potency through various tests, including the analysis of cell surface markers (for identification), cytokine production capacity (for function and potency) and sterility (Riviere and Sadelain, 2017). We evaluated surface and intracellular markers associated with T-cell activation, inhibition, memory and cytokine signaling. Our study demonstrated that the surface protein analysis data indicated that the T-cells remained functional after expansion. Analysis was conducted at the pre-transfer and post-harvest stages of the expansion in static and bioreactor systems. The CD4 to CD8 T-cell ratio indicated the final product’s quality (Figures 5A, B).

The harvested cell products significantly impacted the distribution of CD4^+^ T helper (Th) and CD8^+^ T cytotoxic (Tc) cells. Initially, the CD4:CD8 ratio was higher in the seed culture at (5:1), but after cultivation in various bioreactor systems, it decreased to (2:1) across all three donors. Generally, the IL-2 supplemented culture over 12 days generated more CD8^+^ T-cells. The stirred-tank Applikon bioreactor produced the highest CD8^+^ T-cell population for donor 3 (day 5 vs. day 12: 17.53% ± 1.74% vs. 38.67% ± 7.01%). In comparison, the lowest CD8^+^ seed was observed from the biaxial rotary Tisxell system (day 5 vs. day 12: 16.10% ± 0.56% vs. 18.05% ± 4.90%) for donor 1. T-cell subsets were defined by CD197, CD45RA and CD45RO expression into naïve T-cells (TN: CD45RA+ CD197+), effector T-cells (TE: CD45RA+ CD197−), central memory T-cells (TCM: CD45RO+ CCR7+) and effector memory T-cells (TEM: CD45RO+ CCR7−). Monitoring memory cell types is crucial for quality control because they can be influenced by exogenous factors such as stimulation and cell expansion. Additionally, the donors’ age and health conditions can alter the ratio of memory T-cells to naïve T-cells and central memory T-cells can differentiate into effector memory T-cells. The expression of three memory T-cell receptors—CCR7, CD45RO and CD45RA in T-cells derived from three donors using various bioreactor systems was investigated. In this approach, CD4^+^ and CD8^+^ T-cells exhibited different distributions of naïve, memory and effector phenotypes (Figure 5A), with variations observed between donors and across culture expansions.

Both CD4^+^ and CD8^+^ generated differing profiles of cell subtype populations. The predominant fractions were the effector memory phenotype, comprising 55.83% ± 5.72% to 89.50% ± 2.15% of the harvested CD4^+^ T-cell populations, while the effector T-cells showed differentiation ranging from 23.67% ± 8.80% to 83.77% ± 2.46%. High total cell frequencies were obtained from both the Applikon and Tisxell systems. Naïve T-cells and central memory T-cells were minimally expressed in the harvested CD4^+^ T-cell products and were significantly reduced compared to the pre-transfer cell populations. For CD8^+^ T-cells, more significant heterogeneity was observed in the naïve/memory populations among the harvested products, with variations noted between donors and bioreactors. Notably, TE cells were predominantly expressed with frequencies ranging from 55.83% ± 4.05% to 79.57% ± 2.46%, on which the highest yield was generated from the Tisxell system. The rest of the subpopulations produced similar profiles for all three donors.

The exhaustion status of T-cells was evaluated in the harvested cell products by flow cytometry, examining the expression of PD-1, LAG-3, and TIM-3 (Figure 5B). The culture expansion systems demonstrated an upregulation of all inhibitory signaling receptors on pre-transfer (day 5), indicative of the limited proliferative capacity of the cultivated T-cells among the CD4^+^ subpopulations. The proportion of CD4^+^ PD-1+ T-cells significantly decreased during expansion, dropping from an average of 40.94% (ranging from 22.57% to 59.57%) pre-transfer to an average of 3.28% (ranging from 0.37% to 8.16%) at harvest for all donors. The lymphocyte activation gene three protein (LAG-3) expression, an inhibitory receptor associated with T-cell exhaustion and the release of suppressive signals, decreased from 76.53% to 86.33% on Day 5 to 11.53% to 30.57% on Day 12.

We assessed the intracellular dynamics of T-cells by quantifying the levels of key cytokine classes (IL-2, IFN-γ, and IL-4) at both pre-transfer and harvest stages (Figure 5C). There was a high background of IL-2 and IL-4 produced by pre-expansion of T-cells, but the harvested cell products exhibited significantly lower background levels across all donors and bioreactor systems. An elevated T-cell population of IL-4 was observed for donors two and three compared to the respective static cultures. IFN-γ was marginally expressed (5%–10%) of the total population, corresponding to the low expansion of CD8 memory cell subtypes (Figure 5C).

## Discussions

The bioreactor plays a crucial role in ensuring successful cell culture. Therefore, temperature, dissolved oxygen (DO) and pH must be maintained within precise limits to optimize production volume. Although there are generally defined operational windows for cell culture based on cellular requirements and sensitivities (Baudequin et al., 2021), there is some debate regarding the impeller’s importance in promoting mixing and gas mass transfer versus its potential for causing cell damage (Sieck et al., 2013).

The applications of single use bioreactors have significantly influenced the manufacturing of biopharmaceuticals, leading to their broad utilization in various aspects. The flexibility, cost efficiency, and the minimized risk of cross-contamination are some factors that have facilitated the adoption. Single use bioreactors are effective in culturing human T-cells; thus, their use in cellular immunotherapy is appropriate since it enhances the control of shear stress and the conditions for proliferation (Carswell and Papoutsakis, 2000). The growth of cell culture bioprocessing reflects the innovative technological practices that have made it possible to improve and scale up the systems. This development makes single use technologies relevant in modern bioprocessing (O’Brien and Hu, 2020). Moreover, integrating hybrid and disposable facilities provides a rational alternative to traditional and disposable systems. Ravise et al. (2009) describe the potential and challenges of the systems by emphasizing the operational benefits and the considerations that arise from the management of waste and material conservation using single use bioreactors. Overall, these insights illustrate the transformative impact of single use bioreactors on the biopharmaceutical industry, driving advancements in the production of complex biological therapies.

Stirred-tank bioreactors have emerged as a promising solution for the scalable production of cell therapies, addressing many of the limitations associated with traditional planar culture systems. These bioreactors offer a controlled environment for cell growth, significantly reducing manual processes’ variability and contamination risks (Abraham et al., 2018). Their ability to support suspension cultures is particularly advantageous for the large-scale expansion of mammalian cells, including neural precursor cell aggregates, as demonstrated by Gilbertson et al., who achieved successful scale-up using computer-controlled systems (Gilbertson et al., 2006). Additionally, Bohnenkamp et al. (2002) highlighted the efficacy of stirred-tank bioreactors in cultivating human T-lymphocytes on a clinical scale, underscoring their potential to enhance the efficiency and consistency of cell therapy production. By leveraging automation and precise control over culture conditions, stirred-tank bioreactors facilitate more reliable and cost-effective manufacturing processes, paving the way for broader adoption of cell-based therapies.

The advantages of using a biaxial rotation bioreactor were reported previously in several papers. Computational Fluid Dynamics (CFD) has been applied to enhance understanding of flow phenomena in scaffolds within the Tisxell system, considering its direct effects on fluid flow processes and cellular responses such as cell attachment, migration and proliferation (Singh et al., 2005; Hutmacher and Singh, 2008). Leferink et al. (2015) demonstrated that starting with a static culture of human fetal MSCs (hfMSCs) on synthetic polymeric scaffolds and subsequently transferring and culturing the scaffolds in a biaxial rotating bioreactor enhanced the uniformity of cell and extracellular matrix distribution and increased the overall cell count. Moreover, the relative mRNA expression levels of markers for stemness and differentiation remain stable, while static culture exhibits variations in several markers for stemness and differentiation. The biaxial rotating bioreactor provided a uniform distribution of hfMSCs, facilitating studies on the fate of MSCs in additively manufactured scaffolds without triggering undesired differentiation. The Tisxell system presented herein provided a fully-sealed environment with pre-installed oxygen and pH control devices compared to the other bioreactors. The closed bioreactor system helps minimize the contamination risk and maintain sterility. It is essential for producing cells and tissues for use in regenerative medicine, where product purity and safety are critical.

WAVE bioreactors have become an increasingly popular choice for producing cell therapies, offering several advantages over traditional bioreactor systems. The disposable nature of WAVE bioreactors simplifies the setup and cleanup processes, reducing the risk of cross-contamination and making them ideal for clinical applications (Singh, 1999). These bioreactors utilize wave-induced agitation, providing gentle mixing that supports the growth and expansion of delicate cell types. Donia et al. (2014) demonstrated a streamlined protocol for producing clinical-grade tumor-infiltrating lymphocytes using WAVE bioreactors, highlighting their efficiency and ease of use in a clinical setting. Additionally, Meng et al. (2018) showed that WAVE bioreactors could rapidly expand cells to a clinical scale for tumor immunotherapy, emphasizing their scalability and potential for high-throughput applications. The adaptability and efficiency of WAVE bioreactors make them a foundational tool in the clinical manufacturing of CAR T-cells, as they support consistent and high-quality cell production (Wang and Rivière, 2016). These characteristics position WAVE bioreactors as a critical technology in advancing cell-based therapies.

The feasibility of scale-up in bioreactor systems for future allogeneic cultures will depend on several factors, including the specific type of bioreactor, the nature of the cultured cells, and the desired output. Stirred-tank bioreactors are frequently used in the mass production of cells and biological products. Scale-up in these systems is generally feasible, but challenges such as maintaining homogeneous conditions and controlling shear forces must be carefully considered. Biaxial rotary reactors are suitable for culturing sensitive cells, but their scalability is limited due to mechanical constraints. WAVE bioreactors have demonstrated scalability and the ability to generate low shear forces, making them a promising option for allogeneic cultures. However, the high cost and specialized knowledge required for operation may limit their widespread adoption. Overall, while all three bioreactor systems have the potential for scale-up of allogeneic cultures, thoughtful attention to the exact needs and constraints of the application is necessary to determine the most appropriate system (Stephenson and Grayson, 2018).

In conclusion, we have demonstrated in developing a scale-up platform for high-quality human T-cell production. To our knowledge, this study is the first to demonstrate the feasibility of culturing human primary T-cells in an agitated system (i.e., stirred-tank, biaxial rotary reactors). There is no agreement on the most effective bioreactor method to produce T-cells in sufficient quantities for clinical use. Overall, all three systems showed achievable proliferation rates equivalent to industry standards. It was shown that T-cells cultured in stirred-tank bioreactors under dynamic conditions facilitated increased cell densities that do not negatively affect cell quality. Substantial quantities of functional T-cells at a therapeutic dose scale were generated within a short timeframe. Further, scale-up studies should be performed using the optimized processes to expand cell yield and composition. A perfusion-based feeding strategy should be evaluated to determine if higher cell densities can be achieved in a minimal timeframe using the Applikon system. It highlights the process that could be applied to future CAR-T cell production.

## Data Availability

The raw data supporting the conclusions of this article will be made available by the authors, without undue reservation.
